# Low Skeletal Muscle Mass in the Lower Limbs Is Independently Associated to Knee Osteoarthritis

**DOI:** 10.1371/journal.pone.0166385

**Published:** 2016-11-10

**Authors:** Sang Yoon Lee, Hee Joon Ro, Sun G. Chung, Si Hyun Kang, Kyung Mook Seo, Don-Kyu Kim

**Affiliations:** 1 Department of Physical Medicine & Rehabilitation, Chung-Ang University College of Medicine, 102, Heukseok-ro, Dongjak-gu, Seoul 156–755, South Korea; 2 Department of Rehabilitation Medicine, Seoul National University College of Medicine, 101 Daehang-ro, Jongno-gu, Seoul 110–744, South Korea; West Virginia University School of Medicine, UNITED STATES

## Abstract

**Objectives:**

It has been reported that low skeletal muscle mass correlates with knee osteoarthritis in obese individuals. This study aimed to investigate whether lower limb skeletal muscle mass is independently associated with knee osteoarthritis in the general population.

**Materials and Methods:**

This cross-sectional study used public data from the Fourth and Fifth Korean National Health and Nutrition Examination Survey. Subjects included 4924 community-dwelling adults aged ≥50 years (821 subjects with knee osteoarthritis and 4,103 controls). Skeletal muscle mass index (SMI) was calculated from the appendicular skeletal muscle mass measured by dual energy X-ray absorptiometry. Independent effects of total and lower limb SMI values on knee osteoarthritis were determined using odds ratios (OR) adjusted for age, sex, obesity, total femur bone mineral density, serum vitamin D level, diabetes mellitus status, and physical activity on multivariate logistic regression analysis.

**Results:**

The adjusted logistic regression model revealed that older age, female sex, and obesity were significantly associated with knee osteoarthritis. A higher serum vitamin D level was also positively correlated with knee osteoarthritis (OR, 1.015; 95% CI, 1.003–1.027; P = 0.010). Although total SMI was not significantly associated with knee osteoarthritis (OR, 0.976; 95% CI, 0.946–1.007; P = 0.127), a low lower limb SMI had an independent effect on knee osteoarthritis (OR, 0.941; 95% CI, 0.900–0.983; P = 0.006).

**Conclusions:**

Low skeletal muscle mass in the lower limbs but not in the whole body was independently associated with knee osteoarthritis.

## Introduction

Knee osteoarthritis (OA), the most common joint disorder in the elderly, results in several disabling conditions [1]. Physical activity level, smoking, diabetes mellitus (DM), and obesity are modifiable risk factors and could be the targets of knee OA prevention [2, 3]. Above all, obesity is one of the best documented risk factor for knee OA [4]. Although overweight status itself could trigger knee OA by mechanical loading [5], one study reported no dose-response relationship between body mass index (BMI) and the progression of knee OA [6]. They suggested that a progressively higher BMI was not accompanied by a progressively increasing rate of joint space narrowing. Because the total body mass includes fat tissues that secrete various adipokines [7] that induce the pathophysiology of knee OA as well as skeletal muscles, separation of these two components from the total body mass is needed to predict the real risk factors of knee OA [8].

Evidence of muscle weakness in knee OA exists [9] but has not been fully explained by the effects of aging. Glass *et al*. revealed that quadriceps weakness was associated with an increased risk of knee pain worsening by a 5-year longitudinal cohort study of 4,648 female knee joints [10]. However, one reported that the anatomical cross sectional area (CSA) of the quadriceps muscles appeared to be more sensitive for detecting the progression of knee OA than isometric knee extensor strength from a 2-year follow-up study [11]. They pointed out that strength is more variable than CSA to be correlated with knee OA. Although CSA of thigh muscle might give objective and quantitative data of lower limb muscle mass [12], it is just an extremely localized portion of the whole extremity and requires computed tomography with high-dose radiation exposure. However, few studies have investigated the association between total lower limb muscle mass and knee OA.

Karlsson *et al*. reported that patients with knee OA have a phenotype with higher fat mass and lower lean body mass and suggested that subjects who have lower lean body mass will lower the joint-protective ability [13]. Low skeletal muscle mass also correlates with knee OA in the obese [14]. Those authors showed that sarcopenic obesity was more closely associated with radiographic knee OA than was non-sarcopenic obesity after comparing four large groups of patients who were roughly divided by both sarcopenia and obesity. Although these studies have some clinical relevancies, lean body mass or skeletal muscle mass index (SMI), which were used in these studies to measure the skeletal muscle mass in patients with knee OA, contains both lower and upper limb muscle mass.

One systematic review article criticized that the literature lacks basic science studies and that it could not be concluded whether sarcopenia has a direct effect on OA [15]. The more direct effect of sarcopenia on knee OA should be shown by a skeletal muscle mass analysis that focuses on the *lower* limbs as well as analyses that include a multivariate regression model because there are several risk factors of knee OA. Therefore, this study aimed to investigate whether skeletal muscle mass of the lower limbs is independently associated with knee OA in the general population. We hypothesized that skeletal muscle mass of the lower limbs would show higher correlation with knee OA than that of whole body in the subjects with knee OA.

## Materials and Methods

### Study population

This is a cross-sectional study using the public data from the IV-3, V-1, and V-2 Korean National Health and Nutrition Examination Survey (KNHANES) conducted from 2009 to 2011, which is a national surveillance system for assessing the health and nutritional status of Koreans conducted by the Korea Centers for Disease Control and Prevention. This study included 27,982 community-dwelling people. We analyzed target subjects aged ≥50 years who had entirely undergone physical examinations (height, weight, waist circumference, presence of knee pain, and International Physical Activity Questionnaire (IPAQ)), medical history study about chronic diseases (such as hypertension, DM, stroke, heart disease, and malignancies), and laboratory tests (about serum 25-hydroxyvitamin D [25(OH)D] level, dual energy X-ray absorptiometry (DEXA) of the entire body, and plain radiography of the bilateral knee joints). Patients who had a medical history of rheumatoid arthritis were excluded. A total of 4,924 subjects (2,179 men and 2,745 women) were ultimately included in the current study. Subjects with knee OA were defined as those who had knee pain with a Kellgren/Lawrence grade ≥2 on the radiographic study [16]. Among the total of 4,924 subjects, 821 had knee OA and 4,103 had not. This study is an analysis of public data from KNHANES and therefore ethical approval was not required for the study.

### Anthropometric data and serum vitamin D level

From anthropometric data (height, weight, and waist circumference), BMI was calculated as the individual's body mass divided by the square of one’s height (kg/m^2^). Serum 25(OH)D levels were measured at the Neodin Medical Institute using blood samples. All samples were tested by radioimmunoassay (1470 WIZARD gamma-counter; PerkinElmer, Finland) within 24 hours after collection. Vitamin D insufficiency was defined as a serum 25(OH)D level of 20 ng/mL according to the Endocrine Society clinical practice guideline [17].

### Knee OA assessment

The presence of knee pain was defined by asking the participants whether they had experienced arthralgia of the knee for >1 month of the past 3 months. Plain X-rays of the weight-bearing bilateral knee joints were obtained from the antero-posterior and lateral aspects with 30° of knee flexion. The radiographic findings relating to knee OA were rated using the Kellgren/Lawrence grading system by two musculoskeletal radiologists with concordant grades accepted. When there was a difference of one grade between the two radiologists’ assessments, the higher grade was accepted [14].

### Body composition and physical activity measurements

A DEXA (DISCOVERY-W, Hologic, MA) was used for the body composition analysis. Fat mass, lean mass, and bone mineral density (BMD) were measured by DEXA. Body fat percentage was defined as the percentage of fat mass of the whole body weight. The total femur BMD was selected to correlate with the knee joint [18]. After appendicular skeletal muscle mass (ASM) was calculated by the sum of the lean mass in the bilateral upper and lower limbs, sarcopenia was diagnosed based on SMI (ASM/total body weight as %) [19] and the cut-off value for defining sarcopenia in Korean (SMI 29.53% in men and 23.20% in women) was calculated from two standard deviations less than the mean value for sex-specific young and healthy individuals (age, 20–39 years) from the 4th KNHANES [20]. The lower limb SMI value was calculated as the skeletal muscle mass of both lower extremities normalized by total body weight (%). The cut-off value of obesity was defined as a BMI ≥27.5 kg/m^2^, which is associated with increased mortality rates in Asians [21] and sarcopenic obesity was defined as the condition in which criteria of both obesity and sarcopenia were satisfied. The short version of the IPAQ in Korea (IPAQ-K) [22], which measures health-related physical activity in populations, was used to measure each subject’s current physical activity. Continuous activity scores expressed as total metabolic equivalents (METs) were calculated as follows: (daily minutes of walking × days per week with walking × 3.3) + (daily minutes of moderate-intensity activity × days per week with moderate-intensity activity × 4.0) + (daily minutes of vigorous activity × days per week with vigorous activity × 8.0) [23].

### Statistical analysis

Independent T-tests were used for mean comparisons of age, anthropometric data, body composition with sarcopenia variables, walking frequency, and total METs from the IPAQ-K between knee OA and control groups. Comparisons of the prevalence of DM, hyperlipidemia, obesity, sarcopenia, and sarcopenic obesity between the two groups were conducted using the Chi-square test. The independent effects of SMI and lower limb SMI on knee OA were determined using multivariate logistic regression models, both unadjusted and adjusted for seven key prognostic factors: age, sex [14], obesity [24], BMD [25], DM [26], serum 25(OH)D level [27], and physical activity [1] (METs/week). The adjusted model was developed through backward elimination with a significance level of 0.1 to enter and 0.05 to stay. We also evaluated possible multiple collinearities between covariates by correlation analysis and collinearity statistical tests (tolerance and variance inflation factor tests), as suggested for regression analysis. PASW Statistics 18 (SPSS Inc., Chicago, IL) was used for all analyses. *p*-values < 0.05 were considered to be statistically significant.

## Results

### Subjects’ characteristics

Subjects with knee OA had a higher mean age (68.2±8.1 *vs*. 62.2±8.5 years, *p*<0.001), female sex ratio (3.56 *vs*. 1.05, *p*<0.001), BMI (25.1±3.4 *vs*. 23.8±3.0 kg/m^2^, *p*<0.001), obesity prevalence (22.4% *vs*. 10.6%, *p*<0.001), body fat percentage (33.0±7.5% *vs*. 28.7±7.8%, *p*<0.001), and prevalence of DM (18.3% *vs*. 12.5%, *p*<0.001) than the control group. In addition, the knee OA group showed significantly lower BMD of the total femur (0.79±0.14 *vs*. 0.85±0.14 g/cm^2^, *p*<0.001), ASM (15.6±3.6 *vs*. 17.3±4.1 kg, *p*<0.001), SMI (25.8±4.0% *vs*. 28.0±4.2%, *p*<0.001), lower limb SMI (19.4±2.8% *vs*. 21.1±2.9%, *p*<0.001), and higher prevalence of sarcopenia (33.9% *vs*. 24.4%, *p*<0.001) and sarcopenic obesity (5.2% *vs*. 1.8%, *p*<0.001) than the control group. There were no differences in serum 25(OH)D level and physical activity level between two groups (Table 1).

**Table 1:** Characteristics of subjects by knee osteoarthritis.

	Control (*n* = 4103)	Knee OA (*n* = 821)	P value*
Age (years)	62.2±8.5	68.2±8.1	<0.001
Sex (*n*)	M 1999: F 2104	M 180: F 641	<0.001^†^
Waist circumference (cm)	83.1±8.8	86.3±9.4	<0.001
BMI (kg/m^2^)	23.8±3.0	25.1±3.4	<0.001
Obesity (*n* (%))	433 (10.6)	184/821 (22.4)	<0.001^†^
Diabetes mellitus (*n* (%))	513 (12.5)	150/821 (18.3)	<0.001
Hyperlipidemia (*n* (%))	455/4103 (11.1)	107/821 (13.0)	0.110
Serum Vitamin D (ng/ml)	19.1±7.0	19.3±7.4	0.581
Vitamin D insufficiency (*n* (%))	2449/4103 (59.7)	482/821 (58.7)	0.602
BMD of total femur (g/cm^2^)	0.85±0.14	0.79±0.14	<0.001
Total fat mass (kg)	17.4±5.4	20.0±6.2	<0.001
Body fat percentage (%)	28.7±7.8	33.0±7.5	<0.001
Total lean body mass (kg)	43.4±8.7	40.1±7.5	<0.001
ASM (kg)	17.3±4.1	15.6±3.6	<0.001
ASM/Ht^2^ (kg/m^2^)	6.6±1.1	6.4±0.9	<0.001
SMI (ASM/Wt, %)	28.0±4.2	25.8±4.0	<0.001
Lower limbs SMI (%)	21.1±2.9	19.4±2.8	<0.001
Sarcopenia (*n* (%))	1001/4103 (24.4)	278/821 (33.9)	<0.001^†^
Sarcopenic obesity (*n* (%))	75/4103 (1.8)	43/821 (5.2)	<0.001
Walking frequency (days/week)	4.0±2.7	4.0±3.0	0.962
Physical activity (10^3^ MET/week)	2.77±4.24	2.61±4.18	0.320

OA, osteoarthritis, WC, waist circumference; BMI, body mass index; BMD, bone mineral density; ASM, appendicular skeletal muscle mass, SMI, skeletal muscle mass index; MET, metabolic equivalent of task

*independent T-test and

^†^Chi-square test for group differences.

### Independent effects of skeletal muscle mass on knee OA

On multivariate logistic regression, no significant collinearity was identified for any of the covariates in statistical tests of collinearity. Unadjusted regression analysis showed that older age, female sex, obesity, lower BMD of the total femur, DM, and total and lower limb SMI were significantly associated with knee OA (*p*<0.001 for all). The adjusted model with total SMI showed that a higher serum 25(OH)D level was also positively correlated with knee OA (OR, 1.015; 95% CI, 1.003–1.027; P = 0.010). However, total SMI did not show an independently significant association with knee OA (OR, 0.976; 95% CI, 0.946–1.007; P = 0.127). Finally, the adjusted model with lower limb SMI revealed a significant negative correlation with knee OA (OR, 0.941; 95% CI, 0.900–0.983; P = 0.006) (Table 2).

**Table 2:** Unadjusted and adjusted odds ratios of knee osteoarthritis.

	Unadjusted OR* (95% CI)	P value	Adjusted OR† (95% CI)	P value	Adjusted OR‡ (95% CI)	P value
Age	1.083 (1.074–1.093)	<0.001	1.096 (1.084–1.108)	<0.001	1.095 (1.083–1.107)	<0.001
Sex (female)	3.383 (2.837–4.036)	<0.001	4.215 (3.367–5.278)	<0.001	3.235 (2.416–4.332)	<0.001
Obesity (BMI 27.5)	2.448 (2.021–2.966)	<0.001	2.451 (1.971–3.046)	<0.001	2.216 (1.763–2.787)	<0.001
BMD of total femur	0.034 (0.020–0.060)	<0.001	2.271 (1.037–4.977)	0.040	2.161 (0.984–4.745)	0.055
Serum vitamin D	1.003 (0.993–1.014)	0.570	1.015 (1.003–1.027)	0.010	1.017 (1.005–1.028)	0.005
Diabetes mellitus	1.564 (1.281–1.910)	<0.001	1.268 (1.018–1.578)	0.034	1.237 (0.993–1.542)	0.058
Physical activity (MET)	0.991 (0.972–1.009)	0.320	1.015 (0.996–1.035)	0.116	1.018 (0.999–1.038)	0.066
SMI	0.876 (0.860–0.894)	<0.001	0.976 (0.946–1.007)	0.127	-	
Lower limbs SMI	0.815 (0.792–0.838)	<0.001	-		0.941 (0.900–0.983)	0.006

OR, odds ratio; CI, confidence interval; BMI, body mass index; BMD, bone mineral density; SMI, skeletal muscle mass index; MET, metabolic equivalent of task

*Unadjusted odds ratios by logistic regression analysis.

Adjusted odds ratios by multivariate logistic regression analysis; adjusted for all other variables in model with SMI† and both legs SMI‡.

**-** Not included in the final adjusted model.

## Discussion

A total of 821 patients with knee OA and 4103 subjects without knee OA were compared by anthropometric data, body compositional variables (including lean body mass, total fat mass, and sarcopenic indices), existence of DM or hyperlipidemia, serum 25(OH)D level, and physical activity calculated as METs per week using the IPAQ-K. Age, female sex, obesity, and the presence of DM showed positive correlations with knee OA, while BMD of the total femur, total SMI, and lower limb SMI had negative correlations with knee OA from the unadjusted analysis. Although total SMI did not show a significant association with knee OA, a low lower limb SMI value had an independent effect on knee OA on adjusted multivariate regression analysis. To our knowledge, this is the first study to reveal that skeletal muscle mass in the lower limbs is independently associated with knee OA in the general population.

### Lower limb muscles and knee OA

Several studies suggested that knee extensor muscle weakness is associated with knee OA. From a case-control study with 26 female participants, one reported that quadriceps weakness might contribute to knee OA onset and progression although hamstring muscle strength and gait pattern were not associated with the disease [28]. Becker *et al*. even suggested that quadriceps muscle dysfunction may be an etiologic factor of the pathologic changes of knee OA from the maximal voluntary contraction comparisons of 32 patients who underwent partial meniscectomy and the control group [29].

Dannhauer *et al*. reported that the anatomical CSA of the quadriceps muscles appeared to be more sensitive for detecting the progression of knee OA than isometric knee extensor [11]. From a 4.5-year long-term analysis of 117 patients with symptomatic knee OA, Wang Y *et al*. also suggested that increased vastus medialis CSA was associated with reduced knee pain and beneficial structural changes of the knee joint [30]. Although CSA of thigh muscle might give objective and quantitative data of lower limb muscle mass [12], it is just an extremely localized portion of the whole extremity and requires computed tomography with high-dose radiation exposure. Furthermore, knee joint might also be affected by mechanical loading both above and below the joint [31]. However, one study have investigated the association between total lower limb muscle mass and knee OA.

To our knowledge, only one study has revealed the correlation between lower limb muscle mass and knee OA. They suggested the total lean body mass of lower limb was significantly lower in women with knee OA [32]. However, they simply compared the mean muscle mass values between the knee OA and control groups and many confounding factors such as age and obesity were not considered in statistical analyses. Furthermore, they used a bioelectrical impedance analysis to measure one's body composition, which has many fundamental limitations compared with DEXA [33]. In the current study, total lower limb muscle mass measured by DEXA was revealed as an associated factor with knee OA, whereas total appendicular skeletal muscle mass was not. Because most of the possible confounders that could affect knee OA were adjusted, we can strongly suggest that lower limb muscle mass is an independent factor of knee OA. Therefore, sarcopenic variables containing muscle mass of both upper and lower limbs would not be related factors on knee OA. Instead, lower limb muscle mass, which can influence knee joint mechanics, might be an important factor for predicting and evaluating knee OA.

Although the direct effect of muscle mass on joints of lower limb should be examined to lower limb muscle mass separately, most studies have used total appendicular limb mass which contained both upper and lower limb muscles. Lee *et al*. reported that low skeletal muscle mass, which defined from skeletal muscle mass of 4 extremities, correlates with knee OA in the obese [14]. Recently, one also suggested that patients with knee OA have a proportionally lower lean body mass, not lower ‘lower’ limb lean body mass [13]. While skeletal muscle mass might be shown similar patterns between upper and lower limbs in an individual, they have been studied to have different aging processes. After comparing muscle thickness, torque, and power between the elbow and knee muscles (flexors and extensors), Candow *et al*. reported that lower limb muscles are affected more by aging than are upper limb muscles [34]. In our study, only lower limb muscle mass showed a negative correlation with knee OA, unlike total limb muscle mass. Therefore, limb-specific muscle mass examinations should be needed to study the effect of skeletal muscles on a specific joint.

### Vitamin D and knee OA

Several reports have suggested that a lower serum vitamin D level might be a risk factor of OA. From a longitudinal study (average, 8 years) with 237 subjects, one reported that lower serum levels of 25(OH)D may be associated with incidental changes in hip OA characterized radiographically by cartilage loss [35]. The authors hypothesized that lower serum 25(OH)D levels might increase metalloproteinase enzyme activity for destroying articular cartilage. Ding *et al*. also revealed that serum 25(OH)D levels are associated with decreased knee cartilage loss from a cohort study of 880 subjects [27]. They suggested that this phenomenon was observed at the whole range of 25(OH)D levels rather than predefined cut-off points. From these cumulative studies, Sanghi *et al*. investigated a randomized controlled trial with 107 patients to delineate the therapeutic effect of oral vitamin D supplement on knee OA and reported significant pain reduction and functional improvement after 12 months of drug administration [36].

However, results from the current study were beyond our expectations. Higher serum 25(OH)D levels showed a positive correlation with the presence of knee OA from the adjusted regression model (OR, 1.017; 95% CI, 1.005–1.028). Because age [37], obesity [38], and physical activity [39] which might affect the serum vitamin D level, were also included in the final regression model, a higher serum level of 25(OH)D could be an independent associated factor of knee OA. One recent clinical trial showed that vitamin D supplementation did not reduce knee pain or cartilage volume loss in patients with symptomatic knee OA whereas it elevated 25(OH)D plasma levels [40]. From a longitudinal cohort study, Felson *et al*. also insisted that low vitamin D levels were not directly associated with knee OA worsening [41]. Interestingly, two animal studies suggested that hypervitaminosis D can result in muscle weakness, which could influence knee OA [42, 43]. Therefore, further basic studies and clinical trials are needed to reveal whether a higher level of vitamin D is harmful to the joint cartilage and skeletal muscles.

### Limitations of this study

There are several limitations in this study. Firstly, it is difficult to establish a causal relationship between knee OA and skeletal muscle mass at the present stage because of the genuine limitations of cross-sectional studies. Furthermore the effect size of lower limb muscle mass on knee OA was not large. After converting OR to Cohen’s *d* which indicates the effect size [44], *d* was 0.567 and it was medium effect [45]. Therefore, further longitudinal studies or controlled trials are needed to reveal the causality. Secondly, lower limb strength was not measured in this study. Several working groups for sarcopenia have recommended the measurement of muscle strength as well as muscle mass and they defined “pre-sarcopenia” as the status with decreased muscle mass with normal muscle strength [33, 46]. Although one study suggested that muscle mass might be more sensitive than muscle strength for evaluating the progression of knee OA [11], muscle strength correlates more closely with physical function [47]. Segal *et al*. also reported that thigh muscle mass does not appear to confer protection or worsening knee OA and insisted that knee extensor neuromuscular activation and muscle physiology were more important than muscle mass itself [48]. Instead of measuring muscle strength, we calculated and compared the physical activity derived from the IPAQ-K, although it cannot be a perfect substitute for muscle strength. Thirdly, we cannot discriminate and analyze unilateral knee OA cases because KNHANES did not provide the laterality of knee pain or knee radiographic findings. Therefore, we did not separate both lower extremities and 'lower limb SMI' value was also calculated as the skeletal muscle mass of both lower extremities. Although bilateral knee OA is more frequent than unilateral knee OA (34.1% vs. 12.5% in the Beijing Osteoarthritis Study) [49], separating both sides should be needed for more accurate analysis.

## Conclusions

This study showed that a low level of skeletal muscle mass in the lower limbs correlated with the presence of knee OA and the association was independent through the adjusted regression analysis. However, the appendicular skeletal muscle mass of the whole body did not show an effect on knee OA. Although it is a cross-sectional study, this is the first clinical report to implicate that knee OA could be independently influenced by specific lower limb muscle mass. Further longitudinal studies or controlled trials are needed to reveal the causal relationship of this phenomenon.
